# Ovarian reserve parameters and response to controlled ovarian stimulation in infertile patients

**DOI:** 10.12669/pjms.35.4.753

**Published:** 2019

**Authors:** Qurat ul Aman Siddiqui, Sagheera Anjum, Fatima Zahra, Sarim Muhammad Yousuf

**Affiliations:** 1 Dr. Qurat ul Aman Siddiqui, FCPS. Associate Professor, Department of Obstetrics and Gynaecology, Liaquat National Hospital and Medical College, Karachi, Pakistan. Australian Concept Infertility Medical Centre,; 2 Dr. Sagheera Anjum, FCPS Assistant Professor, Department of Obstetrics and Gynaecology, Liaquat College of Medicine and Dentistry and Darul Sehat Hospital, Karachi Pakistan; 3 Dr. Fatima Zahra, FCPS Assistant Professor, Department of General Medicine, Liaquat College of Medicine and Dentistry and Darul Sehat Hospital, Karachi Pakistan; 4 Sarim Muhammad Yousuf, Ziauddin Medical University (3^rd^ Year MBBS),

**Keywords:** Ovarian reserve, AMH, AFC, FSH, Female age

## Abstract

**Objective:**

To determine the ovarian reserve parameters in patients presenting for IVF and intracytoplasmic sperm injection (ICSI) treatment and its association with the number of follicles retrieved and number of oocyte retrieved and fertilized.

**Methods:**

A retrospective cross sectional study was conducted at Australian Concept Infertility Medical Centre from January 2017 to August 2017. Around 120 couples presenting to infertility clinics selected for IVF and ICSI with Females (25-45) had their FSH, AMH and AFC done. After ovulation induction, its response was determined by number of follicles retrieved, quality of oocytes retrieved or fertilized and inseminated. SPSS version 20 was used for the purpose of data analysis.

**Results:**

The median age of the patients was 34 (29-38) years. A moderate negative correlation of age and FSH levels was observed with quality of oocytes, Number of oocyte inseminated, number of oocyte fertilized and number of follicle restored. However, a positive correlation of AMH and AFC levels were found with quality of oocytes, Number of oocyte inseminated, number of oocyte fertilized and number of follicle restored. The correlation of AMH levels with number of oocyte inseminated (rho 0.729, p-value <0.001), number of oocyte fertilized (rho 0.721, <0.001) and number of follicle restored (rho 0.723, p-value <0.001) were found strongly correlated.

**Conclusion:**

Our study concluded that AMH and AFC have a strong correlation with number of follicles restored and number of oocytes retrieved whereas FSH and age has a weak correlation with the number of follicles restored and number of oocytes retrieved.

## INTRODUCTION

Ovarian reserve is a measurement of the ovarian follicular pool^1^ and determines the capacity of the ovary to produce oocytes that are capable of fertilization. Age is one of an important factor for ovarian reserve quality and quantity determination.^1^ Follicular stimulating hormone (FSH) is the test widely used for ovarian reserve.^2^ Serum Antimullerian hormone (AMH) is also a reliable marker that reflects the acyclical ovarian activity. This marker is operator independent and there is strong correlation in predicting ovarian reserve,^3^ it is a glycoprotein secreted by the granulosa cells of the developing ovarian follicles. It is a serum marker for the measurement of ovarian reserve and can be done on any day of the menstrual cycle.^4^ The antral follicle count (AFC) is done via transvaginal ultrasound (TVS) to measure ovarian reserve and is done on the 3^rd^ day of menstrual cycle.^5^ An antral follicle count (AFC) of less than 3 to 7 indicates a decrease in the ovarian reserve and subsequently poor ovarian response in in vitro fertilization (IVF) cycles.^6^ Day 3 FSH is a marker of hypothalamic pituitary ovarian axis with increasing age the amount of FSH increase due to poor response by the ovary therefore increasing FSH reflects diminished ovarian reserve.^7^ Assessment of women with poor ovarian reserve before entering an IVF program may help to direct the management of the patient with regards to chances of success to treatment.^8^

Therefore, the objective of this study was to determine the ovarian reserve parameters in patients presenting for IVF and intracytoplasmic sperm injection (ICSI) treatment and to find out the association of ovarian reserve parameters with the number of follicles retrieved, number of oocyte retrieved and number of oocytes fertilized.

## METHODS

A retrospective cross sectional study was conducted at Australian Concept Infertility Medical Centre from January 2017 to August 2017. Institutional ethical approval was obtained (IRB: CMC/RES/158/2017) and written informed consent was taken from the couples involved in this study. Around 120 couples presenting to infertility clinics and selected for IVF and ICSI treatments were included. Female partners aged between 25-45 years having regular menstrual cycles and normal levels of TSH, prolactin and testosterone levels were included in the study. Patients having normal ultrasound pelvis and proven tubal patency by laparoscopy were also included. All the couples having female partners with a history of surgical treatment on the ovary for ovarian cysts, endometriosis, any pelvic surgery, or using any hormonal treatments for last three months were excluded from this study.

All data was collected on a proforma by medical professionals after taking informed consent from the participants. All patients before undergoing IVF cycle had detailed history taking and systemic examination. Information was obtained about age of male and female partner, the type of infertility. Height, weight and body mass index (BMI) of the female partners, and basal serum level of FSH was collected on the second day of menstrual cycle, quantitative assessment for AMH was also collected along with FSH for the convenience of the patient and AFC was measured by transvaginal ultrasound on 3^rd^ to 5^th^ day of menstrual cycle using 2D ultrasound by a sonologist with experience of 5 or more years in an infertility center. Then after ovulation induction using the antagonist protocol the response to ovulation induction was determined by number of follicles restored (number of follicles produced as a result of ovarian stimaulation), quality of oocytes retrieved was noted (analysis of various aspects of oocyte morphology, cytoplasm, zona pellucid and polar body via conventional phase contrast microscoy had generated a criteria of good, moderate and bad quality oocytes), also the oocytes fertilized and inseminated were noted. The outcome of procedure in terms of positive pregnancy test was also noted. SPSS version 20 was used for the purpose of data analysis.

## RESULTS

### Descriptive Statistics

Out of 120 patients, median age of the patients was 34 (29-38) years. Majority of the patients (n=78, 64.7%) had ≤35 years while 42 (35.3%) had >35 years of age. The median BMI of the patients was 28 (25-32) kg/m^2^. There were 76 (66.7%) non-obese and 38 (33.3%) obese patients. The median infertility years was 7 (4-10). There were 70 (58.3%) patients with ≤7 years of duration of infertility and 50 (41.7%) patients with >7 years of duration of infertility. Primary infertility type was observed in majority (n=82, 68.3%) and secondary infertility type in 35 (29.9%) patients.

### Comparison of type of procedure with baseline characteristics

The infertility type was the only variable found significantly associated with type of procedure (p-value <0.001). ICSI was found significantly higher among patients with primary infertility type than that of secondary infertility type whereas Intra Cytoplasmic Sperm Injection – Gender Selection (ICSI-GS) and Frozen Embryo Transfer (FET) procedures were found significantly higher among patients with secondary type of infertility. Other variables like Age (p-value 0.723), BMI (p-value 0.701), and duration of infertility (p-value 0.069) were found insignificant.

### Comparison of follicle restored with baseline characteristics

The follicle restored was found significantly associated with age (p-value <0.001) and duration of infertility (p-value 0.026). While BMI (p-value 0.641) and type of infertility were found to be statistically insignificant (p-value 0.896).

### Correlation of oocytes with ovarian reserve parameters

A moderate negative correlation of age and FSH levels were observed with quality of oocytes, number of oocyte inseminated, number of oocyte fertilized and number of follicle restored. However, a positive correlation of AMH and AFC levels were found with quality of oocytes, number of oocyte inseminated, number of oocyte fertilized and number of follicle restored. The correlation of AMH levels with number of oocyte inseminated (rho 0.729, p-value <0.001), number of oocyte fertilized (rho 0.721, <0.001) and number of follicle restored (rho 0.723, p-value <0.001) were found strongly correlated. (Table-II).

**Table I:** Comparison of type of procedure, follicle restored and outcome with baseline characteristics of the females (n=200).

Variables	Total	Type of Procedure				Follicle Restored			

		ICSI(n=106)	ICSI-GS(n=10)	FET(n=4)	p-value	≤10(n=56)	11-20(n=38)	>20(n=26)	p-value
	
	n (%)	n (%)	n (%)	n (%)		n (%)	n (%)	n (%)	
Age, years	*34 (29-38)*	34 (29-37)	32 (25-39)	35 (32-39)	0.723^‡^	36 (32-39)	32 (27-36)	30 (25-33)	<0.001
≤35	78 (64.7)	70 (89.7)	6 (7.7)	2 (2.5)	0.77	25 (32.1)	28 (35.8)	25 (32.1)	<0.001
>35	42 (35.3)	36 (85.7)	4 (9.5)	2 (4.8)		32 (76.2)	9 (21.4)	1 (2.4)	
BMI, kg/m2 (n=114)	*28 (25-32)*	28 (25-32)	28 (26-34)	27 (27-27)	0.701	28 (25-33)	28 (25-31)	28 (25-31)	0.641
Non-obese	76 (66.7)	69 (90.8)	6 (7.9)	1 (1.3)	0.703	32 (42.1)	25 (32.9)	19 (25)	0.721
Obese	38 (33.3)	34 (89.5)	4 (10.5)	0 (0)		18 (47.4)	13 (34.2)	7 (18.4)	
Infertility, years	*7 (4-10)*	7 (4-10)	3 (2-7)	4 (2-19)	0.069	8 (4-11)	6 (4-11)	5 (3-8)	0.096
≤7	70 (58.3)	59 (84.3)	8 (11.4)	3 (4.3)	0.259	26 (37.1)	24 (34.3)	20 (28.6)	0.026
>7	50 (41.7)	47 (94)	2 (4)	1 (2)		30 (60)	14 (28)	6 (12)	
Type of infertility (n=117)									
Primary	82 (68.3)	79 (96.3)	3 (3.7)	0 (0)	<0.001	37 (45.1)	27 (32.9)	18 (22)	0.896
Secondary	35 (29.9)	25 (71.4)	7 (20)	3 (8.6)		17 (48.6)	10 (28.6)	8 (22.9)	

*Abbreviation:* **ICSI:** intracytoplasmic sperm injection, **ICSI-GS:** intracytoplasmic sperm injection –gender selection, **FET:** frozen embryo transfer

All patients with >30 kg/m^2^ were considered as obese and ≤30kg/m^2^ BMI were considered as non-obese

†MANN-Whitney U test,

‡ Kruskal Wallis Test, Chi-square test applied, p-value <0.05 was taken as significant

**Table II:** Correlation of oocytes with ovarian reserve parameters.

	Good quality oocyte		Moderate quality oocyte		Bad quality oocyte		Number of oocyte inseminated		Number ofoocyte fertilized		Number of follicle restored	

	rho	p-value	rho	p-value	rho	p-value	rho	p-value	rho	p-value	rho	p-value
Age, years	-0.312	<0.001	-0.308	<0.001	-0.351	<0.001	-0.344	<0.001	-0.362	<0.001	-0.360	<0.001
FSH, mIU/ml	-0.258	0.005	-0.246	0.007	-0.180	0.050	-0.307	<0.001	-0.275	0.002	-0.355	<0.001
AMH, ng/ml	0.598	<0.001	0.581	<0.001	0.496	<0.001	0.729	<0.001	0.721	<0.001	0.723	<0.001
AFC	0.419	<0.001	0.370	<0.001	0.415	<0.001	0.502	<0.001	0.501	<0.001	0.491	<0.001

**AMH:** Antimullerian Hormone, **AFC:** Antral Follicle Count, **FSH:** Follicle Stimulating Hormone Spearman’s test applied, p-value <0.01 was taken as significant

**Table III:** Median difference of general characteristics of patients with respect to the pregnancy outcome.

	Outcome		

	Pregnant (n=25)	Non-Pregnant (n=95)	p-value
Age of female partner, years	31 (25-35)	34 (30-38)	0.007
Age of male partner, years	37 (32-41)	40 (35-45)	0.148
BMI, kg/m2	27 (24-30)	8 (25-32)	0.051
FSH, mIU/ml	6 (4-8)	7 (5-9)	0.099
AMH, ng/ml	4 (2-6)	1 (0.4-3)	<0.001
AFC	12 (10-17)	8 (8-10)	<0.001

All data presented as median (IQR), **AMH:** Antimullerian Hormone, **AFC:** Antral Follicle Count, **FSH:** Follicle Stimulating Hormone, Mann-Whitney U-Test applied, p-value <0.05 was taken as significant

### Comparison of pregnancy outcome with general characteristics

A significant difference of pregnancy outcome was observed with age of female partner (p-value 0.007), AMH level (p-value <0.001), and AFC levels (p-value <0.001). Whereas age of male partner (p-value 0.148), BMI (p-value 0.051) and FSH levels (p-value 0.099) were found to be insignificant.

## DISCUSSION

In human assisted reproduction, there is a variable response to the gonadotropin stimulation by the ovaries therefore it is difficult to predict.^9^ The results of current study demonstrate that AMH has a positive association with the number of follicles restored as well as number of oocytes retrieved. The results also show that AMH has a strong correlation with the number of oocytes inseminated and fertilized. Shembekar et al in a study of 100 patients also demonstrated that high AMH levels correlated with retrieval of more eggs.^10^ Moreover, the study also demonstrated high chances of successful fertilization and insemination with raised AMH levels.^10^ This could be due to the fact that AMH is a representation of the number of oocytes left in the ovary thereby resulting in a consistent measurement between the menstrual cycles. Due to this fact it can be assumed that AMH is considered as a first line investigation for evaluation of ovarian reserve.^4^,^11^

The results of our study also demonstrate that FSH has a weak correlation with the number of follicles restored and number of oocytes retrieved. This contrasts with AMH that has a strong correlation with retrieval of oocytes. Our results are compatible to the ones reported by Jamil et al.^12^ and Parveen et al.^13^ Parveen et al compared the diagnostic accuracy of AMH with FSH for the assessment of ovarian reserve. Their study demonstrated that AMH has greater sensitivity for the assessment of ovarian reserve.^13^ Studies conducted in Netherlands consider AMH as a marker of ovarian aging.^14^,^15^

Our study results also show that there is a negative correlation of FSH levels with number of oocyte inseminated, number of oocyte fertilized and number of follicle restored. According to a study conducted by Islam et al, FSH and ovarian volume do not correlate with the ovarian response^16^ whereas Thum et al. concluded that women with high basal FSH respond well to stimulation and produce good number of oocytes giving them an equal chance to become pregnant as compared to normal women of their age.^17^

Antral follicles are measured by the means of TVS. AFC of 8-10 is considered as a predictor of normal response whereas more than 14 considered as a good predictor of hyper response.^18^,^19^ The results of this study showed a strong positive correlation of AFC with the number of follicles restored, number of oocytes retrieved and the quality of oocytes retrieved. These results are comparable with a study that also showed that AFC has a positive correlation with number of oocytes retrieved.^20^ Usmani et al has reported that ovarian volume and AFC is significantly reduced in older age group.^21^ Therefore, it can be assumed that AFC is a reliable indicator of ovarian reserve. A similar finding concluded in a study conducted in India in 2018 .^22^ American society of reproductive medicine (ASRM) and European Society of Human Reproduction and Embryology (ESHRE) in their publication for best practice have shown that AMH has the best sensitivity and specificity for measurement of ovarian response to controlled ovarian hyperstimulation (COH).^23^

Our study showed that age is associated with number of follicles restored. Moreover, the study results showed that moderate negative correlation exists between age and ovarian reserve a similar finding in a study conducted by Hessein in 2015.^24^ Jehan et al also concluded that sub-fertile women the age greater than or equal to 35 years had a reduced potential of fertility.^25^

### Limitations of the study

This was a single institute study and was conducted on a small sample size. Moreover, only one cycle of ovulation induction was observed.

Despite these limitations we acknowledge certain strengths of this study that it effectively evaluates ovarian reserve and its response to stimulation in couples attending the infertility clinics in the reproductive age group and selected for IVF treatment. The outcome was measured in response to ovulation induction. All the biomarkers done in the study participants were done in the follicular phase to avoid inconvenience for the patients and avoiding variation in results due to the phase of the menstrual cycle. It is recommended that further prospective multicenteric studies on larger sample size be carried out for a more detailed evaluation of ovarian reserve.

### CONCLUSION

Our study concluded that AMH and AFC have a strong correlation with number of follicles restored and number of oocytes retrieved whereas FSH and age has a weak correlation with the number of follicles restored and number of oocytes retrieved.

### Authors Contribution

**QAS & SA:** Conceived, designed and did editing of manuscript

**SA & FZ:** Did data collection, statistical analysis and manuscript writing

**QAS & SA:** Did review and final approval of manuscript

**SMW:** Did data collection.
